# PARP Power: A Structural Perspective on PARP1, PARP2, and PARP3 in DNA Damage Repair and Nucleosome Remodelling

**DOI:** 10.3390/ijms22105112

**Published:** 2021-05-12

**Authors:** Lotte van Beek, Éilís McClay, Saleha Patel, Marianne Schimpl, Laura Spagnolo, Taiana Maia de Oliveira

**Affiliations:** 1 Structure and Biophysics, Discovery Sciences, R&D, AstraZeneca, Cambridge CB4 0WG, UK; lotte.vanbeek1@astrazeneca.com (L.v.B.); marianne.schimpl@astrazeneca.com (M.S.); 2 Institute of Molecular, Cell and Systems Biology, College of Medical, Veterinary and Life Sciences, Garscube Campus, University of Glasgow, Glasgow G61 1QQ, UK; e.mcclay.1@research.gla.ac.uk; 3 Discovery Biology, Discovery Sciences, R&D, AstraZeneca, Cambridge CB4 0WG, UK; saleha.patel@astrazeneca.com

**Keywords:** poly (ADP-ribose) polymerases 1-3, DNA damage response, PARP-DNA binding, ADP-ribosylation, nucleosome remodelling, histone PARylation factor 1, PARP activation

## Abstract

Poly (ADP-ribose) polymerases (PARP) 1-3 are well-known multi-domain enzymes, catalysing the covalent modification of proteins, DNA, and themselves. They attach mono- or poly-ADP-ribose to targets using NAD^+^ as a substrate. Poly-ADP-ribosylation (PARylation) is central to the important functions of PARP enzymes in the DNA damage response and nucleosome remodelling. Activation of PARP happens through DNA binding via zinc fingers and/or the WGR domain. Modulation of their activity using PARP inhibitors occupying the NAD^+^ binding site has proven successful in cancer therapies. For decades, studies set out to elucidate their full-length molecular structure and activation mechanism. In the last five years, significant advances have progressed the structural and functional understanding of PARP1-3, such as understanding allosteric activation via inter-domain contacts, how PARP senses damaged DNA in the crowded nucleus, and the complementary role of histone PARylation factor 1 in modulating the active site of PARP. Here, we review these advances together with the versatility of PARP domains involved in DNA binding, the targets and shape of PARylation and the role of PARPs in nucleosome remodelling.

## 1. Introduction

Poly ADP-ribose polymerase (PARP) enzymes play a key role in a number of cellular processes, such as DNA repair, genome maintenance, and cell death [1,2,3,4,5,6,7]. The best characterised member of the PARP family is PARP1, which was first identified for its role in the recognition and repair of single-strand DNA breaks (SSB) [8,9]. Since then, PARP1 has also been shown to have a role in a number of DNA damage response (DDR) pathways, including base excision repair (BER), homologous recombination (HR), non-homologous end joining (NHEJ), and DNA mismatch repair. Consequently, PARPs have been an attractive target for anti-cancer therapies resulting in the successful development of several PARP inhibitors (PARPi) for ovarian, breast, lung, and pancreatic cancers [10]. This novel class of therapies competes with the native substrate nicotinamide adenine dinucleotide (NAD^+^) for the PARP catalytic site [11,12,13,14,15].

It has been more than half a century since the discovery of PARP1 and the process of poly-ADP-ribosylation (PARylation) that PARP1, and other members of the PARP family, catalyse [1,2,3]. During PARP catalysis ADP-ribose residues are transferred from NAD^+^ onto the target substrate building a poly ADP-ribose (PAR) chain. The building of PAR chains, along with their removal, occurs in all three major divisions of life (eukaryotes, prokaryotes, archaea) [16,17,18]. This review focuses on the human enzymes. The identification of PAR and its structure, and the realisation that PARP1 produces PAR, were key breakthroughs during early PARP research [19,20,21,22]. Studies that followed detailed the purification of PARP1 [23], demonstrated the activation of PARP in response to genotoxic agents [8,24], linked PARP1 to the repair of DNA damage [9], and showed the association of PAR with nucleosome remodelling and enzymes, including PARP1 itself [21,25]. Furthermore, the discovery of PARP2—and other members of the PARP family—was enabled by the generation and characterisation of the *PARP1* knockout mouse [26,27,28].

**Figure 1:**
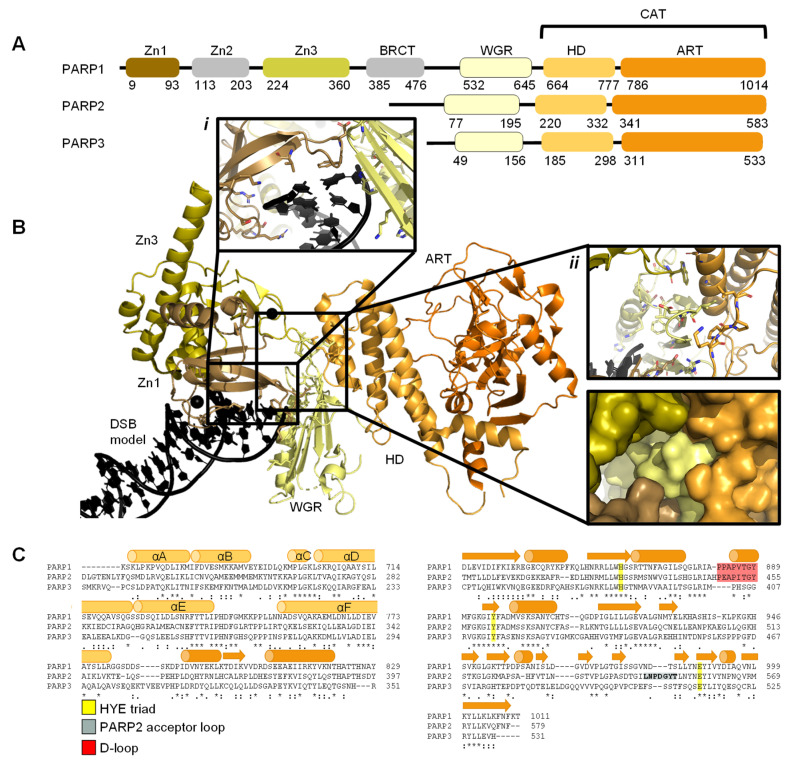
Overview of PARP1, PARP2, and PARP3. (**A**)**.** Schematic overview of the domains of PARP1, PARP2, and PARP3, with amino acid numbers indicating domain boundaries corresponding to Uniprot entries P09874, Q9UGN5, and Q9Y6F1; (**B**)**.** Crystal structure of PARP1 bound to a double-strand DNA molecule representing one end of a double-strand break (DSB) (Protein Data Bank (PDB) entry 4DQY [34]). Domain colours correspond to A. Inset *i*: interactions of Zn1 and WGR with a DSB model. Inset *ii*: interdomain interactions between Zn1, Zn3, WGR, and HD shown as cartoons (top) and surface representations (bottom). All figures in this review containing protein structures were generated in PyMOL [43]; (**C**)**.** Structure-based sequence alignment of CAT domains of PARP1, PARP2, and PARP3 was generated in PyMOL [43]. Sequence alignment was generated in Clustal Omega [44] provided by EMBL-EBI [45]. Secondary structure is represented by cylinders as α-helices and arrows as β-sheets, respectively. Sequence conservation is shown, where an asterisk represents fully conserved, a colon represents strongly similar properties and a period represents weakly similar properties. The conserved histidine, tyrosine, glutamic acid (HYE) triad is shown in yellow, the PARP2 acceptor loop in grey, and the D-loop in red.

The PARP family consists of 17 proteins in humans. PARP1, PARP2, PARP5A, and PARP5B are capable of synthesising PAR chains [1,2,3]. Most other members in the PARP family catalyse the addition of single ADP-ribose units and are therefore classified as mono ADP-ribosyltransferases (MARs). PARP1, PARP2, and PARP3 are DNA-dependent enzymes [4]. PARP1, the largest of the three, is approximately 116 kDa [29] and comprises of six independently folded domains: the N-terminus consists of three zinc (Zn) finger domains Zn1, Zn2, and Zn3; this is followed by the auto-modification domain, which contains the BRCA1 C-terminus (BRCT) fold and mediates protein–protein interactions; adjacent to this is the tryptophan, glycine, arginine (WGR) motif; whilst at the C-terminus sits the catalytic (CAT) domain (Figure 1) [30,31,32,33]. Four of these domains (Zn1, Zn2, Zn3, and WGR) have been shown to bind DNA (Figure 1B) [34]. The CAT domain is the most conserved across the PARP family (Supplementary Figure S1) and comprises of the helical subdomain (HD) and the ADP-ribosyl transferase (ART) subdomain (Figure 1C) [30,31]. Interestingly, PARP2 and PARP3 only share the C-terminal regions (WGR and CAT domains), yet they are able to regulate the mechanism of DNA-induced activation via local destabilisation of the HD. It has been proposed that human PARP2 contains an additional N-terminal DNA and/or RNA-binding domain [35,36,37]: the SAP motif named after the proteins in which it was found, SAF/Acinus/PIAS [38]. This motif occurs one to four times at the N-terminus of plant PARP2 [39,40] and is a putative DNA-binding four-helix bundle [41]. However, Riccio et al. (2015) [42] report that although the N-terminus of PARP2 is important for PARP2 activation on SSBs, the N-terminal region of human PARP-2 is intrinsically disordered [42]. Therefore, here the SAP motif is not included in the domain overview of PARP2 (Figure 1A).

Early structures of individual PARP domains were first published in the late 1990s for PARP1 [46,47,48], and early 2000s for PARP2 [49]. The first crystal structure of the human PARP1 CAT domain bound to a small molecule inhibitor was published by Kinoshita and co-workers in 2004 [50], and subsequently, for PARP2 by Karlberg et al. (2010) [51] and for PARP3 by Lehtiö and colleagues [52]. Thereafter, many more structures have been published of PARP1-3, often of individual domains and short truncated forms of the proteins. To date there have been no reported structures of full-length PARP1. However, the full-length structure of PARP2 and histone PARylation factor 1 (HPF1) in complex with a nucleosome was recently determined by cryo-electron microscopy (cryo-EM) [53].

There are many reviews focused on specific aspects of PARP biology [54,55] and its specific role in PARylation [56,57], DDR [15,58,59,60], PARPi [61,62,63,64,65], cancer biology [66,67,68], and other disease areas [69,70,71,72]. This review aims to summarise the structural basis of the DNA-dependent PARP1, PARP2, and PARP3 enzymes and their roles in DNA binding, activation, and nucleosome remodelling.

## 2. DNA Damage Recognition by PARP Enzymes

Each cell within an organism faces a high frequency of DNA damage on a daily basis [73,74], due to many endogenous and exogenous causes [75]. Members of the PARP family are key initiators of the DDR pathway amongst other functions [4,6,76]. Repairing damaged DNA is essential for cells to enable successful transcription, maintain genomic stability, achieve cell replication, and survive [75]. The majority of DNA damage within cells is inflicted on just one DNA strand and is known as a single strand break (SSB). These SSBs are often easier to repair given the necessary information is still available on the complementary strand. Different types of SSB damage can occur depending on the process which resulted in the break. For example, a DNA strand can be nicked (both bases are intact but the DNA backbone is broken), the pyrimidine/purine group can be missing resulting in an abasic (AP) site, or a nucleotide can be missing (gap) [73]. Double-strand breaks (DSBs) are more problematic for cells and therefore cells have dedicated pathways to rectify this type of damage. Depending on the cell cycle stage, and the presence of a template, the HR or the NHEJ pathways are initiated [10,77].

PARPs play an essential role as DNA damage sensors, of both SSBs and DSBs [5], and promote DNA repair through recruitment of DNA repair factors. Examples of such PARP1 interactors include: the X-ray repair cross complementing group 1 (XRCC1) [78] acting as a scaffold for DNA SSB repair components (FF9igure 6C); the tyrosyl-DNA phosphodiesterase 1 (TDP1) [79], which removes stalled topoisomerase 1 (TOP1)-DNA complexes (Figure 6D); DNA protein kinase catalytic subunit (DNAPKcs) [80] in the NHEJ DSB repair pathway and variable, diversity, and joining (V(D)J) recombination [81]; the kinase ataxia telangiectasia mutated (ATM) [82,83] in DNA DSB repair; and the nuclease meiotic recombination 11 (MRE11), also in DSB repair (Figure 6B) [84,85].

### 2.1. PARP1 Zn Fingers Bind a DNA Break

**Figure 2:**
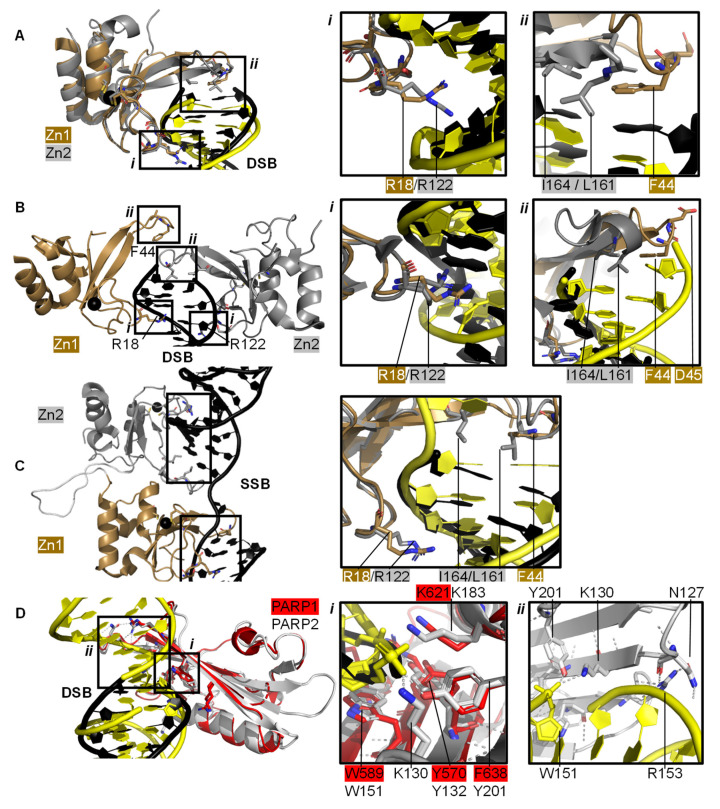
PARP domains in complex with a DNA DSB model (**A**,**B**), SSB (**C**), or DSB (**D**). (**A**) Superposed crystal structures of Zn fingers 1 (Zn1, brown, PDB 3ODA [86]) and 2 (Zn2, silver, PDB 3ODC [86]), RMSD 0.96 Å, in complex with DSB models shown in black for Zn1 and yellow for Zn2. Inset *i* shows the phosphate backbone grip and inset *ii* the base stacking loop; (**B**) Zn1 (brown) and Zn2 (silver) cooperate DSB model binding (PDB 4AV1 [87]). Insets show the superposition of Zn1 and Zn2 with an RMSD of 0.80 Å of the boxed regions. In the superposition, Zn1 and Zn2 binding to a DSB model is shown in black and yellow, respectively, where *i* shows the Zn finger loop with R18/R122 interacting with the major groove of the DSB model and *ii* the base stacking loop with F44/L161/I164; (**C**) Zn1 and Zn2 also cooperate SSB binding (PDB 2N8A [89]). Inset shows the superposition of the boxed regions in Zn1 and Zn2 on a SSB with an RMSD of 0.83 Å, where DNA in yellow binds to Zn1 and DNA in black binds to Zn2; (**D**) Superposition of the WGR domains from PARP1 (red, PDB 4DQY [34]) on a black DSB model and PARP2 (white, PDB 6F5B [90]) bridging a yellow DSB with a 5′ phosphate group (Cα RMSD of 0.89 Å). Inset *i* highlights the superposed WGR PARP1 and PARP2 residues involved in DNA binding. Inset *ii* shows how the WGR of PARP2 interacts with the second DSB using R153.

Individual PARP1 domains Zn finger 1 (Zn1) or Zn finger 2 (Zn2) can both bind to DNA with a Cα root mean square deviation (RMSD) of 0.96 Å, when a double-strand DNA molecule representing one end of a DSB is used as a model for DNA damage (PDBs 3ODA, 3ODC, Figure 2A [86]). Ali and colleagues [87] solved the crystal structure of a protein construct containing both Zn1 and Zn2 connected by their linker on a DSB model (PDB 4AV1). This shows Zn1 and Zn2 cooperate to recognise the DSB model (Figure 2B) yet their binding modes are very similar with a Cα RMSD of 0.80 Å. Zn1 and Zn2 contact DNA at two locations in the phosphate backbone grip (Inset *i* in Figure 2A,B) through R18 (Zn1) or R122 (Zn2) and at the base stacking loop (Inset *ii* in Figure 2A,B) via F44 (Zn1) or L161/I164 (Zn2). In the cooperative interaction these base stacking interactions enhance each other. This happens through hydrophobic protein-DNA interactions mediated by L161/I164 from Zn2 capped with F44 from Zn1. This implicates the Zn finger regions of PARP1 in the binding, and subsequent repair, of DSBs.

Both Zn finger interactions are sequence-independent as demonstrated in Figure 2 and the nuclear magnetic resonance spectroscopy (NMR) structures of Zn finger domains (from PARP1 in absence of DNA overlaid with the protein-DNA complex structures [88]). This shows movement of the base stacking loop of Zn1 upon binding to DNA. Further DNA binding studies imply that the phosphate backbone grip of both Zn1 and Zn2 is key to DNA binding and the base stacking loop contributes modest complementary DNA binding ability. However, only Zn1 is essential for PARP1 activation or PARylation. This “activator” difference is attributed to the sequence diversity between Zn1 and Zn2 surrounding the base stacking loop. A D45A mutation in Zn1, which does not have an equivalent residue in Zn2 in the structure-based sequence alignment, abolishes inter-domain communication within PARP1 leading to PARylation, but this mutation does not prevent DNA binding. This is explained by the fact that D45 in Zn1 points away from the DNA-binding interface and so D45 in Zn1 is essential for inter-domain activation of PARP1 (Figure 2B, inset *ii*). Furthermore, an equivalent residue is absent in Zn2, which explains why Zn2 cannot activate PARP1 on its own [86].

Eustermann et al. (2011) showed that Zn1 and Zn2 can both bind SSBs in structures obtained by NMR [88]. The double domain construct Zn1-Zn2 with a flexible 15 amino acid linker binds the SSB model mainly using its Zn2 in a similar manner to Zn2 alone, confirming that Zn2 binds a SSB in preference to Zn1 [88]. The structural rationale for this was explained by the solution structure of Zn1-Zn2 on a SSB (Figure 2C) [89]. Zn2, the domain with a higher binding affinity for DNA, first senses the DNA SSB and interacts with the more accessible 3’ site. This poises Zn1 for DNA binding to the more cryptic 5′ site thereby twisting the DNA; a binding mode only accessible for SSBs. Zn1 and Zn2 also mediate cooperative binding through a hydrophobic interdomain interface (Figure 2C) [89]. Remarkably, the key residues for interactions with both SSBs and DSBs are conserved and located in the phosphate backbone grip and base stacking loop. This is a testament to the flexibility of Zn finger domains in PARP1 in binding to different DNA break architectures.

The crystal structure of Zn finger 3 of PARP1 (Zn3, PDB 2RIQ) revealed a Zn finger fold different from Zn1/Zn2 [22]. Zn3 uses its N-terminal α-helical region to interact with the minor groove of DSBs [34]. Destabilisation of Zn3 (by the introduction of negative charges in the Zn finger domain structure) resulted in a full-length PARP1 mutant able to bind DNA. However, this was unable to undergo DNA-dependent activation [91] suggesting that a function of Zn3 is to mediate interdomain contacts upon PARP1 activation by DNA binding. This is confirmed by detailed studies into the order of DNA interaction and PARP1 activation [34,89], which describe how DNA binding initiates cooperative interdomain interactions between Zn3 and WGR that support destabilisation of the HD.

The 3.25 Å structure showing the minimal combination of PARP1 domains essential for PARP1 activation on a DSB model comprises Zn1, Zn3, WGR, and the HD-CAT domains [34], even though Zn2 can bind DSB models in an analogous fashion to Zn1 (PDB 4DQY, Figure 1B). Here, Zn1 and Zn3 make adjacent interactions to the ribose phosphate backbone of a DSB. They interact with the WGR domain central to PARP1, which binds the 5′ terminus of the DSB via a range of aromatic and positively charged residues in the central β-sheet and Lys600 in its α-helix, and mediates contacts to the CAT domain (Figure 1B, inset *ii*) [34]. The full-length structure of PARP1 on a DSB remains elusive.

### 2.2. Recognition and Binding of Other DNA Breaks via PARP Domains

PARP2 and PARP3 lack the Zn finger domains yet still recognise specific DNA breaks featuring 5′ phosphate groups and subsequently initiating a DDR [76,90,92,93]. Here, the interaction with DNA is mediated by the WGR domain that is also present in PARP1 (Figure 2D). The structure of PARP2 shows similar binding modes for DNA with (PDB 6F5B) and without (PDB 6F1K) 5′ phosphate group [90] despite preferentially binding damaged DNA featuring a 5′ phosphate group [76]. The key interaction in the PARP2-5′ P-DNA appears to be Y201, which coordinates a hydrogen bond to the 5′ phosphate group in PDB 6F5B (Figure 2D, inset *i*), together with the lysine residues K130 and K183. In both structures the PARP2 WGR bridges a DSB (Figure 2D, inset *ii*)—a feasible model of the physiological interaction of the DNA-binding recruitment domain of DNA damage sensor PARP2.

In addition, the N-terminus of PARP2 bears DNA-binding activity and assists in the activation of PARP2 on SSBs [42]. This region also activates PARP2 in the presence of single-strand RNA molecules, but not double-strand DNA molecules [36].

### 2.3. The PARP Paradox: Finding a Needle in a Haystack

For effective DNA damage sensing, PARP molecules need to swiftly locate damaged DNA within a highly crowded nuclear environment containing lots of intact DNA: the estimated DNA concentration in the nucleus is 100 mg/mL [28,29]. A contributing factor to this is the high nuclear concentration of PARP molecules and in particular PARP1 [29,94], estimated to 7–200 μM from 2 × 10^5^–10^6^ copies of PARP1 per nucleus [29,94], a cell volume between 100 and 10,000 μm^3^ [95] and a ratio of nuclear-to-cell volume of 0.08 [96]. These PARP molecules have a very high binding affinity (low binding constant, K_D_) for damaged DNA, ranging from 3 to 62 nM depending on the PARP molecule and DNA damage site [97,98], as measured by Sukhanova et al. (2019) by atomic force microscopy (AFM). In this study, competitive binding was revealed between different members of the PARP family for similar binding sites on DNA. PARP1 has the highest affinity for nick sites, followed by nucleotide break sites and had the lowest affinity for abasic/apyrimidinic (AP) sites. In contrast, PARP2 had similar affinity for nicked and AP sites and the lowest affinity for nucleotide break sites [98]. This confers an intrinsic difference in DNA binding mechanism, as might be expected with PARP2 lacking DNA-binding Zn domains. AFM binding experiments combined with protein volume measurements provided some insight into the observed oligomeric state of PARP molecules on damaged DNA. PARP1 was mostly monomeric on intact, AP sites and gaps with minute dimer formation on nicked sites, while PARP2 preferentially dimerised on gaps and nicked sites and was monomeric on intact and AP sites [98]. Studies are emerging on the molecular activation mechanism of PARP2 on DSBs (see Section 4).

Another contribution to swift DNA damage sensing comes from the way PARP1 travels along intact DNA—termed the monkey bar mechanism (Figure 6A) [99]. This intersegment transfer mode is estimated to enhance the ability of PARP1 to sense DNA damage threefold, compared to diffusion alone [100] (from stopped-flow experiments of PARP1 WT and a W589A mutant unable to use the monkey bar mechanism) [99,100]. Moreover, Rudolph et al. (2018) showed that since the association of PARP1 with DNA is faster than diffusion PARP1 would struggle to release undamaged DNA once bound [99]. Instead, PARP1 makes good use of excess DNA in the nucleus by dissociating the lower-affinity DNA binding domain WGR from the originally bound DNA-molecule *n*. Due to the high DNA concentration in the nucleus, the WGR domain will bind to another DNA-molecule *n + 1*. This WGR-DNA interaction is primarily mediated through W589 (Figure 2D, inset *i*), which stacks against the ribose sugar of the 5′ strand of DNA. When the stronger DNA-binding Zn finger domains dissociate from DNA *n* they will rapidly bind DNA *n+1*. This transfer enables PARP1 to quickly move between DNA molecules, most of which are undamaged until it finds a DNA break, where the Zn domains make specific interactions with the DNA break [31]. After a conformational change within PARP1, which is most likely pinpointed to the “closure” of the WGR onto DNA that happens on a slower timescale than binding of the Zn fingers to DNA [100], the HD domain gets destabilised and mediates allosteric activation of the catalytic domain of PARP1. This in turn strengthens the affinity of the Zn finger domains for the DNA break [32]. In summary, the tailored affinity of different members of the PARP family for different DNA breaks, swinging between high- and lower-affinity DNA-binding domains, and the sheer number of PARP molecules in the nucleus, are the clue towards finding the DNA strand break needle in the DNA haystack called the nucleus.

## 3. PARP Activation and ADP-ribosylation

PARP1, PARP2, and PARP3 are catalytically active enzymes that covalently modify target proteins [1], DNA [101], and themselves [102]. PARPs use NAD^+^ as a substrate to transfer ADP-ribose unit(s) onto an acceptor [2]. ADP-ribosylation is a prolific and reversible post-translational modification (PTM), which regulates numerous pathways in eukaryotes, prokaryotes, and archaea [16,17,18]. ADP-ribosylation is tightly controlled since excessive activation of PARP1 leads to cell death through NAD^+^ depletion [29,103]. As the “PAR code” has spatial and temporal resolution and relevance, the removal of PAR by poly(ADP-ribose) glycohydrolase (PARG) and other PAR degraders [104,105,106] is equally important for normal cell function, we refer the reader to Harrison et al. (2020) [107] for a detailed review on the PAR balance in the cell.

**Figure 3:**
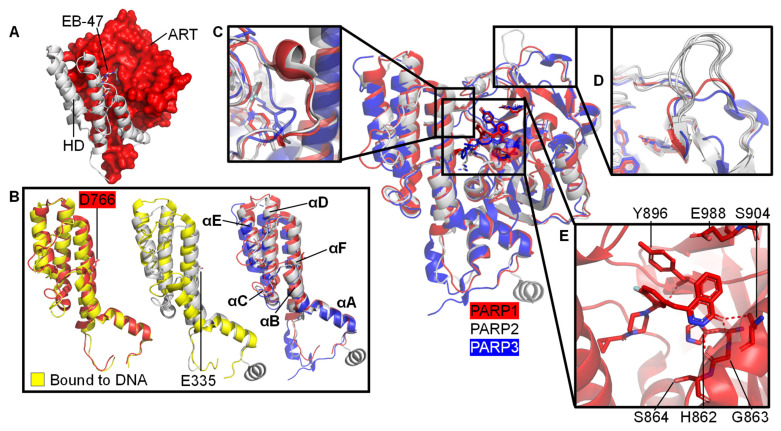
Structural details of the CAT domains of PARP1-3. Central image shows a structural superposition of the ART domains of PARP1 (red, olaparib-bound, PDB 7AAD [109]), PARP2 (white, olaparib-bound, PDB 4TVJ [115]), and PARP3 (blue, ME0354-bound, PDB 4GV2 [116]) with highlighted features. (**A**) A folded HD (white) occludes the NAD^+^ binding pocket in the PARP1 ART domain (red, PDB 7AAB, PARP1 with NAD^+^ analogue EB-47 bound [109]); (**B**) Subtle movement of parts of the HD domains in response to DNA binding after superposition of the ART domain only. Left: HD of apo PARP1 before (red, PDB 7AAA [109]) and after (yellow, PDB 4DQY [34]) DSB model binding. Middle: HD of PARP2 before (white, PDB 4TVJ [115]) and after (yellow, PDB 6X0N [53]) DSB model binding. Right: HDs of PARP1, PARP2, and PARP3 in complex with inhibitors as specified above with annotation of the helices; no DNA binding; (**C**) Close-up of the D-loop of PARP1 and PARP2, which is absent in PARP3; (**D**) The acceptor loop of five representative PARP2 structures (white: PDBs 4ZZX [117], 6X0L, 6X0M, 6X0N [53], 4TVJ [115]) contains a unique insertion compared to PARP1 (red, PDB 7AAD, bound to olaparib [109]) or PARP3 (blue, PDB 4GV2, bound to ME0354 [116]). This is suggested to increase branching of PAR chains by PARP2 [112]; (**E**) Close-up of the NAD^+^ binding pocket of PARP1 (red, olaparib-bound, PDB 7AAD [109]). The catalytic triad involving H862, Y896, and E988 and the residues interacting with PARPi, G863-S864, and S904, are indicated.

The CAT domain consists of the ART domain and the HD bundle. The ART domain possesses the ADP-ribosyl transferase capability with catalytic triad and binding site for PARP inhibitors. The HD regulates the activity of the ART domain: when stably folded against the ART domain, the HD of PARP1-3 occludes the binding pocket and thereby prevents NAD^+^ from binding to the active site (Figure 3A). However, during self-assembly the decrease in entropy allows for enough free state energy to partly destabilise αB and part of αF of HD of the CAT domain, thereby allowing access for NAD^+^ [57,108]. A crystallographic snapshot of this subtle movement is depicted in the superposition of crystal structures of ART domains of DNA-free and DNA-bound PARP enzymes, visualising the introduction of a kink in αF [109] at D766 (PARP1)/E335 (PARP2) (Figure 3B). Section 3.1 goes into this process in more detail including the dynamics measured in helices within the HD.

The D-loop shapes the donor site and interacts with NAD^+^ [110]. It is worth noting that there is no D-loop present in PARP3 (Figure 3C). Perhaps due to its function in mono-ADP-ribosylation (MARylation) only, for example on 5′- and 3’- terminal phosphate residues on DSBs and SSBs [93], whereas both PARP1 and PARP2 are capable of PARylation [102]. The absence of the D-loop may induce differential inhibitor activity compared to PARP1 and PARP2 [111]. The ART domain contains an acceptor site which binds to either the target that is to be ADP-ribosylated or a distal ADP-ribose in an expanding PAR chain. Figure 3D highlights that the PARP2 acceptor loop has six unique inserted residues compared to that of PARP1 and PARP3, increasing its size. This is suggested to be linked to the enhanced ability of PARP2 to generate branched PAR chains, as observed by Chen et al. (2018) [112]. It also contains a donor site, which consists of a nicotinamide binding pocket (HYE conserved triad), a phosphate binding site and an adenine ribose binding site [113]. In PARP1 the conserved triad consists of: the His862 residue, which binds 2’OH of NAD^+^ adenine ribose; the Tyr896 residue, which stacks with the nicotinamide ring; and the Glu988 residue, which forms a hydrogen bond with the 2’OH of the nicotinamide ribose polarising the donor NAD^+^ for nucleophilic attack [4,113,114]. PARP2 and PARP3 have equivalent conserved residues (Figure 3E).

### 3.1. Understanding Activation of a Full-Length PARP1 on a SSB

**Figure 4:**
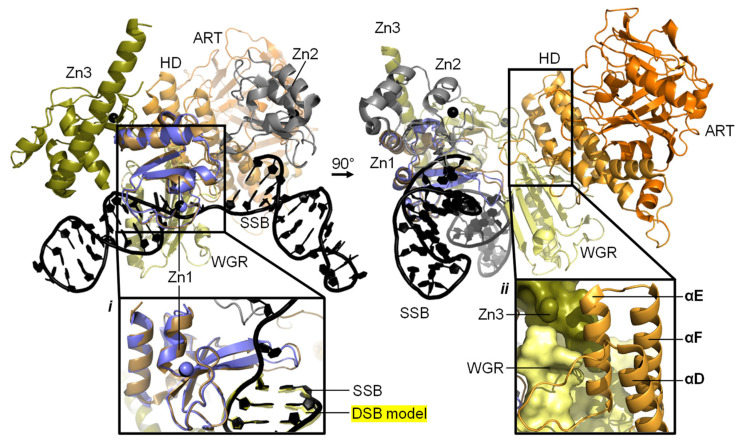
A model of full-length PARP1 on a SSB model. Panels show the resulting composite structure from the superposition of the NMR-structure of Zn1-Zn2 on a SSB model (PDB 2N8A [89]) and the crystal structure of Zn1, Zn3, WGR, HD, and ART on a DSB model (PDB 4DQY [34]) in two views. Domains are annotated, colours correspond to Figure 1 and Zn1 from 4DQY is shown in blue. The composite model was generated through superposing Zn1 and two unpaired and three paired DNA bases of the SSB onto 4DQY, as previously described [1]. Linkers between Zn1-Zn2 and following Zn2 were removed for clarity. Inset *i*: zoom of the superposition of Zn1 (brown from PDB 2N8A and blue from PDB 4DQY) and the superposed DNA bases of the SSB (black, PDB 2N8A) onto the DSB model (yellow, PDB 4DQY). WGR hidden for clarity. Inset *ii*: view of the “landing pad” formed through interface formation between Zn3 (olive) and WGR (pale yellow).

A model composed of the crystal structure of PARP1 domains Zn1, Zn3, WGR, and CAT on a dsDNA molecule representing a DSB, in combination with the solution structure of Zn1-Zn2 on a SSB, gives us an insight into how PARP1 domains might assemble on a SSB (Figure 4). Only through correct placement of Zn1 and Zn2 domains, on a SSB in the observed directionality, do Zn3 and WGR form the “landing pad” assembly (Figure 4, inset *ii*) on which HD can dock and subsequently become partially destabilised to activate PARP1 enzymatic activity [89]. Whilst widely debated, a monomeric PARP1 molecule is sufficient for this auto-activation. This paves the way for auto-modification with PAR [89], as detailed above. This activation mechanism, through local destabilisation, is studied more closely in hydrogen/deuterium exchange-mass spectrometry (HDX-MS) and NMR studies of PARP1 wild-type enzymes and overactive PARP1 mutants. These PARP1 mutants with either the HD completely removed (ΔHD [57]) or a L713F mutation [118] feature increased DNA-independent activity that mimics DNA binding and allosteric activation. Using HDX-MS, the αB helix and part of the αF helix portion of the HD were shown to experience 1000-fold faster exchange [57,109,118]. Additionally, using NMR dynamics experiments significant amide proton exchange was detected in αB, αD, and αF of the HD, while Nuclear Overhauser Effect Spectroscopy (NOESY) experiments showed that the helices were still substantially intact (possibly with the exception of the HD in PARP1 when complexed with the largest inhibitor EB-47) [109]. These experiments provide a nuanced insight into PARP1 protein dynamics at different timescales helping to create a holistic picture of the involvement of the HD in the activation of PARP1.

The regulatory role of the HD subdomain of the CAT domain was confirmed when removal of the HD from PARP1 resulted in a hyperactive enzyme [118]. Figure 3A visualises how a folded HD blocks access to the active site of the CAT domain. In absence of DNA damage no allosteric communication is present and HD is infrequently unfolded, due to low basal dynamics and/or an absence of interactions with other destabilising protein partners [57]. This enables a lower basal rate for PARP1 conversion of NAD^+^ into PAR. As PARP1 is the most abundant protein in the nucleus at approximately 7-200 μM [29,95,96,97], and nuclear [NAD^+^] is estimated to be 400–600 μM [29], PARP could well deplete NAD^+^ in the cell if all PARP was commonly pre-bound to NAD^+^ [57,119].

### 3.2. Targets of ADP-Ribosylation

There have been hundreds of proteins identified as ADP-ribosylation targets within the human proteome [120,121,122,123], such as p53 [124], the p65 subunit of NF-κB [125], several histones [121], and the SSRP1 and Spt16 components of the FACT complex [123,126]. PARP1 protein PARylation activity has been shown to be present in a wide variety of contexts, including SSBs, DSBs, stalled replication forks, and unpaired regions [127]. On target proteins, PARPs have been shown to ADP-ribosylate specific residues; most commonly the acidic residues, but also Arg, Lys, His, Cys, and Tyr [102,122,128]. In addition, in 2016 a new PARP1/2 binding partner was discovered, HPF1, which promotes Ser-ADP-ribosylation [129,130]. Finally, PARPs also directly ADP-ribosylate DNA [101].

On the PARPs themselves there appear to be numerous sites of ADP-ribosylation. Auto-ADP-ribosylation may occur in cis or in trans and this plays a central role in the debate around whether members of the PARP family function as monomers [34,95,101,131,132] or dimers [87,133,134], or perhaps if both mechanisms play a role in different situations [99]. For a more in-depth review on the topic, the reader is referred to Alemasova et al. (2019) [114]. This active debate highlights the interest in PARP research and contributes to the mechanistic understanding of the many roles of PARP enzymes.

One of the earliest reported targets of auto-PARylation was the BRCT fold aptly named the “auto-modification” domain. Reported sites of ADP-ribosylation in the BRCT domain and the following flexible linker in PARP1 include D387, E488, and E491 [135]. However, numerous other sites of auto-ADP-ribosylation sites have been predicted throughout PARP1 [135,136,137]. One mass spectrometry study indicates that in PARP1 the interdomain flexible linkers between the BRCT and WGR domains, and the WGR and HD, are the most prominent ADP-ribosylation targets. In addition, functional domains, such as the D-loop in the CAT domain, and the three Zn fingers were revealed as targets. This study also suggests that PARP2 and PARP3 targets of auto-modification are found within their CAT domains [137].

Most recently, DNA itself has been discovered to be a target of PARylation. PARP1-3 have all been shown to ADP-ribosylate DNA covalently by ADP-ribosylating the 5′ and 3’ terminal phosphates of DNA strand breaks [93,101,138,139]. However, the mechanism and function of DNA ADP-ribosylation is yet to be defined. It is worth noting that the 3’-terminal phosphate residue at a DNA break is the major acceptor site for PARylation by PARP1 at DSBs. This ADP-ribosylation can be highly dependent on the orientation of the DNA strands and the distance between the individual strand breaks [101].

### 3.3. Variation in the Structure and Function of PAR Chains

PAR is a highly negatively charged covalent PTM. There are three stages involved in PAR synthesis: initiation, elongation, and branching [48,140]. During initiation an ADP-ribose monomer is attached to the acceptor residue on the target protein. During elongation a (1″-2′) ribose–ribose α-glycosidic bond is formed between ADP-ribose units. Finally, during branching, a (1‴-2″) ribose–ribose α-glycosidic bond is formed between ADP-ribose units [140]. An important question that was recently touched upon by Aberle et al. (2020) [141] relates to the function of linear and branched PAR chains. PAR chains can vary hugely in length (up to 200 units or 100 nm) [114,142,143] and branching shape. The biological importance of PAR branching was revealed using the PARP1 mutant G972R that yields short and “under-branched” PAR chains. Consequently, cells with this PARP mutant displayed decreased cell viability and increased genotoxic stress and delayed recruitment of downstream PARP1 interaction partner XRCC1 [141]. The formation of branched PAR chains emerges from an interplay between PARP1 and PARP2. Whilst PARP1 mostly initiates the PAR chain, the N-terminal region of PARP2 recognises PAR chains, DNA, and RNA and performs secondary PARylation including branching [112]. PARP1 also introduces branches into PAR chains, although PARP2 seems to be the main source of branching. It is hypothesised that this difference has an underlying structural reason, where the acceptor binding site of PARP2 (white) is extended compared to that of PARP1 (red) or PARP3 (blue) (Figure 3D), although the molecular mechanism of binding a wide range of acceptor molecules currently remains elusive. The importance of this is exemplified by variation in affinity of PAR binding factors, such as H1, p53, and xeroderma pigmentosum group A (XPA) for various PAR chains [141,144,145].

Another intriguing question regarding PAR extension concerns the extension mechanism. This relates to whether units are either added close to the CAT domain (proximal model [146]) or to the far end of the extending PAR chain (distal model [147]). Currently the distal model is most widely supported. For an elaborate review of these two models the reader is referred to a review by Alemasova et al. (2019) [114]. The distal model raises a question: how does PARP1 add units to a chain that is ~100 nm away from its catalytic centre? This brings us back to the debate of whether PARPs act as monomers or dimers.

## 4. PARP, HPF1, and Nucleosome Remodelling

PARP1-3 have been shown to be almost ubiquitous in the cellular response to DNA damage. However, for multi-protein DNA repair complexes to assemble at the DNA damage site chromosome relaxation must occur, and PARylation is one of the first PTMs employed at these sites. On detection of DNA damage the enzymatic activity of PARP1 may be activated [34] and chromatin remodelling factors recruited. ADP-ribosylation has long been linked to chromosome decondensation. In 1982, Poirier et al. showed that PARP1 ADP-ribosylated histone H1, resulting in a relaxed chromatin formation that could be visualised using electron microscopy [148].

### 4.1. The Role of HPF1

**Figure 5:**
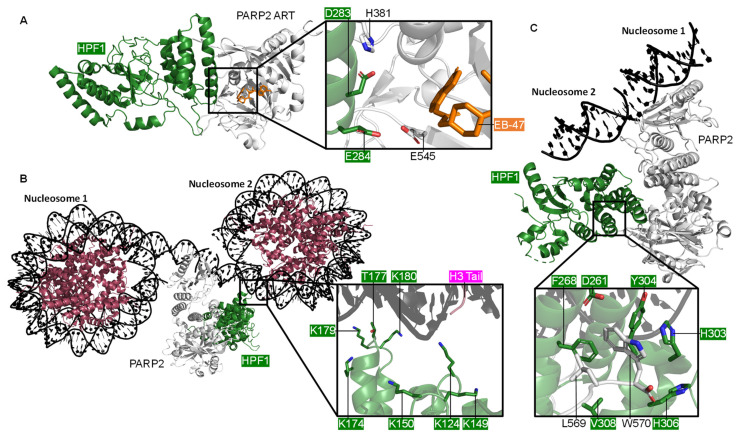
The PARP2 and HPF1 Complex. ***(*A**) The crystal structure of HPF1 and PARP2 CATΔHD (ART subdomain), showing the composite catalytic site with NAD^+^ analogue EB-47 bound (PDB 6TX3 [150]); (**B**) The cryo-EM structure of PARP2 and HPF1 bridging one side of a DSB between two nucleosomes, showing the key positively charged residues of HPF1 that interact with the nucleosome (PDB 6X0N [53]); (**C**) A closer look at the cryo-EM structure of the PARP2, HPF1, and nucleosome complex (PDB 6X0M [53]), showing the interactions between the C-terminus of PARP2 and the C-terminal domain of HPF1.

Recently HPF1 has emerged as an accessory factor linked to PARP1 and PARP2 ADP-ribosylation of histones in the context of DNA lesions. It was previously thought that PARPs primarily ADP-ribosylate the acidic residues [149]; although HPF1/PARP binding has been shown to promote Ser-ADP-ribosylation [129,130]. The first structure published of PARP2 and HPF1, a crystal structure by Suskiewicz et al. (2020) [150], shows the proteins forming a composite enzyme where each partner contributes catalytic residues to the substrate binding pocket. D283 of HPF1 and H381 of PARP2 stabilise the interaction whilst E284 of HPF1 contributes to the catalytic site (Figure 5A). The PARP2 C-terminal residues L569 and W570 interact with the HPF1 C-terminus (Figure 5, inset *ii*). A mutational analysis showed the role of individual residues in this composite enzyme. HPF1(E284A) does not produce detectable Ser-ADP-ribosylation with WT PARP1 indicating that E284 is necessary for efficient catalytic deprotonation in the Ser-ADP-ribosylation reaction [150].

The PARP1 modular domain architecture and flexibility has continuously hindered structural studies with most labs being forced to study various truncations of the protein. As previously mentioned, the Pascal group crystallised four of the six domains of PARP1 [34] (Figure 1B); although a full-length structure has not yet been obtained. Perhaps the “resolution revolution” that cryo-EM is currently undergoing may provide a solution for this problem [151,152,153]. Recently the first full-length cryo-EM structure of a PARP enzyme was published. This structure shows PARP2 and HPF1 in complex with a nucleosome [53]. Positive residues of the N-terminal domain of HPF1 interact with the nucleosomal DNA (Figure 5B). The biggest surprise from this structure was that a single PARP2/HPF1 complex can bridge two nucleosomes and that the pair of PARP2/HPF1 complexes that bridge either side of the DSB do not appear to interact. Of note, the arginine (R140) of the conserved WGR triad of PARP2 is the main point of contact with the second nucleosome. However, in the case of the PARP1 crystal structure R591 of the WGR triad contacts Zn1 and not the DSB model.

HPF1 has been suggested to only bind the active form of the PARP enzymes. Binding is either inhibited by the HD subdomain or promoted by DNA or NAD^+^ binding to PARP [150]. In both the crystal [150] and the cryo-EM [53] structures of the HPF1 and PARP2 complex, PARP2 is in an active conformation. In the crystal structure, the HD autoinhibitory subdomain is missing, whereas in the cryo-EM structure PARP2 is DNA bound and the rearrangement of αB of the HD enables HPF1 binding. It is worth noting that structurally PARP1-2 are relatively well conserved (see Supplementary Figures S1 and S2) and interactions between HPF1 and PARP2 are located at some of the most conserved sites. As was shown in the crystal structure, the PARP2 C-terminal residues L569 and W570 interact with the HPF1 C-terminus (Figure 5C), the equivalent residues are L1013 and W1014 in PARP1. It is interesting that in 55 of the 63 PDB entries containing the CAT domain of PARP1 the construct lacks the final two C-terminal residues, which may be vital for interactions for HPF1. The PARP1 CAT appears to have been consistently truncated because a successful protocol for crystallisation of this domain was established and repeated; however, it may be worth noting going forward the potential importance of these residues when looking at binding partners.

### 4.2. Histone Remodelling

Ser ADP-ribosylation is one of many dynamic modifications that shape histones and it is reversible by ADP-ribose hydrolase 3 (ARH3) [154,155]. The core histones can be ADP-ribosylated and their N-terminal tails have been proposed to be the primary acceptors of Ser ADP-ribosylation [156]. The histone tails are known to be frequently and extensively covalently modified by phosphorylation, methylation, acetylation, and ubiquitylation [157]. Combinations of these modifications, including Ser ADP-ribosylation, act collaboratively to dictate chromatin dynamics in contexts, such as DNA repair, transcription, and replication. The majority of studies examining histone modifications have overlooked Ser ADP-ribosylation as its presence has only been uncovered in recent years. Mutational studies argue that the basic residue preceding the Ser is as important as the Ser residue itself [156,158]. The primary site of H3 Ser ADP-ribosylation in vivo was shown to be Ser10 [149]. Acetylation of Lys9 in H3 was shown to significantly inhibit Ser10 ADP-ribosylation. Meanwhile phosphorylation of Ser10 completely inhibits ADP-ribosylation of the same residue and vice versa [158,159]: both of these proposals are indicative of the interplay between PTMs on the histone proteins that dictate nucleosome dynamics. Although originally identified in histone modification Ser ADP-ribosylation is widespread and strongly enriched in proteins involved in processes including DNA repair [149]. It has also been shown to be prominent in the response to oxidative stress [123]. There is a growing body of evidence showing the wide extent of serine ADP-ribosylation, mostly localised on nuclear proteins [121,122,123]. Ser ADP-ribosylation can be inhibited by the PARP inhibitor, olaparib [123] evidencing that PARPs are a major driver of ADP-ribosylation.

In the past, ADP-ribosylation studies have been hindered by the charged and heterogeneous nature of these PTMs. Nevertheless, in recent years mass spectrometry has come to the forefront in categorising protein ADP-ribosylation [160,161] and electron transfer dissociation (ETD) fragmentation has been proposed as the optimum technique for reducing ADP-ribose fragmentation [162]. Other methods include using hydroxylamine to convert acidic ribosylated side chains to a hydroxamic acid derivative [120]. High resolution mass spectrometry was recently used to identify thirty Asp and Glu ADP-ribosylation sites on the core histones (H2A, H2B, H3, and H4) and also H1, which binds linker DNA between adjacent nucleosomes, following dimethyl sulphate induced DNA damage [163].

### 4.3. Other Interaction Partners of PARP1

One of the biggest questions raised by the manipulation of PARP1/2 by HPF1 is whether there are numerous proteins that direct PARP enzymatic activity in various contexts. A potential candidate is Nicotinamide Nucleotide Adenylyltransferase 1 (NMNAT-1), which appears to direct PARP1 ADP-ribosylation to the acidic residues in adipogenesis [164].

**Figure 6:**
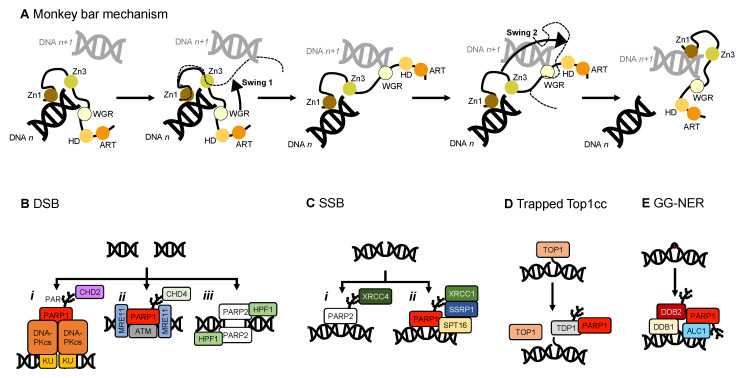
A schematic showing how PARP1 recognises DNA damage, and examples of the roles of PARP1 and PARP2 in initiating DDR and chromatin remodeling. (**A**) The monkey bar mechanism [99]; (**B**) PARP1/2 initiating DSB repair. *i.* PARP1 interacts with DNA-PK [79] and recruits the chromatin remodeler CHD2 [174] in the canonical NHEJ pathway. *ii.* PARP1 plays a role in the alternative NHEJ pathway, interacting with MRE11 (part of the MRN complex, [84]), ATM, and recruiting the CHD4 subunit of the NuRD complex [166,167]. *iii.* PARP2 recognises a DSB along with HPF1 [53,150]; (**C**) PARP1/2 initiating SSB repair. *i.* PARP2 plays a role in recognising SSBs and recruiting XRCC4 [175]. *ii.* PARP1 can also recognise SSBs and recruit XRCC1 [78,176], and subsequently the FACT complex (SSRP1 and SPT16; [176]); (**D**) PARP1 recruits TDP1 to release trapped TOP1 cleavage complexes (Top1cc) [79]; (**E**) PARP1 is involved in global genome nucleotide excision repair (GG-NER), recruiting DNA damage-binding protein 1 and 2 (DDB1/2) and the chromatin remodeler ALC1 [177].

Although ADP-ribosylation of histones at the sites of DNA damage, and subsequent relaxation, is evident, the intermediate steps are yet to be determined. One of the most likely theories is that on PARP recognition of DNA damage ADP-ribosylation recruits chromatin remodelling factors whilst initiating the DDR (Figure 6). One such complex is Facilitate Chromatin Transcription (FACT) [126]. PARylation at SSBs has been shown to dictate the recruitment of histone chaperones [165]. In the context of a DSB the chromatin surrounding the break is rapidly and transiently PARylated recruiting the nucleosome remodelling and deacetylase (NuRD) complex [166] most likely via its Chromodomain-helicase-DNA-binding protein 4 (CHD4) subunit which contains a PAR binding motif in its N-terminal region [167]. The NuRD complex can conduct ATP-dependent chromatin remodelling, histone deacetylation, recruit DNA repair and checkpoint factors, and assist transcriptional silencing [168]. Therefore, PARP1 may act to recognise DNA damage through its enzymatic modification of the DNA break and recruit necessary factors to decondense the surrounding chromatin enabling access of other DNA repair factors to the site of damage.

It has previously been shown that inactive PARP1 compacts chromatin [94], whereas PARylated nucleosomes result in decondensed chromatin, as shown in electron micrographs [148]. Inhibiting PARylation has been proposed to prevent chromatin relaxation at sites of DNA damage, due to the lack of the recruitment of a protein named Amplified in liver cancer 1 (ALC1) [169]. ALC1 is a DNA helicase, and a member of the SNF2 family of chromatin remodellers, which is localised to sites of DNA damage through the binding of PAR chains (Figure 6E) [170]. Recently ALC1 has moved to the forefront of small molecule PARPi research as it can underlie resistance to PARPi treatment [171]. ALC1 overexpression is common in tumours and increases resistance to PARPi. The loss of ALC1 in *BRCA* mutant cells has been shown to sensitise them to olaparib whilst significantly reducing the half maximal inhibitory concentration (IC_50_). In fact, in cells with engineered PARPi resistance through restoration of the HR pathway ALC1 deficiency has been proposed to reinstate PARPi sensitivity [172]. The structure of ALC1 in complex with a nucleosome was recently published revealing ALC1 regulation by both an acidic patch on nucleosomes and its macro domain binding to PAR chains at sites of DNA damage [173]. Given the emerging evidence of the role of ALC1 in PARylation dependent chromatin relaxation and PARPi resistance, it has been proposed as a possible target for combination therapies.

Given that currently most structures, functions, and mechanisms of the possible interaction partners of PARP remain elusive there are likely many more interactors. This poses an exciting area of future work that could shed more light on the versatility of PARP and with it, potential new therapeutics.

## 5. Concluding Remarks

This review outlines what we currently know of the structure and activation mechanisms of PARP1-3, from DNA binding and activation, to binding partners and inhibition. Despite PARP research being well-established, the ubiquitous nature of PARP enzymes results in the continuous evolution of novel directions in the field. The pursuit of the PARP1-3 structures has progressed along with structural biology. This is exemplified by the recent publication of a high-resolution structure of full-length PARP2 using cryo-EM. Although the full-length PARP1 structure remains elusive, the most promising approach may be binding PARP to DNA and an interacting protein, helping overcome the hindrances of its modular domain architecture and flexibility in the apo form.

The structures of PARP1-3 presented in this review are prime examples of how the activation mechanism of a protein can be uncovered using integrative structural biology. Possible future directions in the field include: structural studies of HPF1 with other PARP family members; translating the PAR code on new targets of ADP-ribosylation; the extension mechanism of PAR; and structures of PARP1-3 bound to various interactors. Overall, the study of PARPs and ADP-ribosylation is a complex and exciting field exploring the fundamentals of cellular function and producing invaluable therapies for cancer treatment.

## Supplementary Materials

The following are available online at https://www.mdpi.com/article/10.3390/ijms22105112/s1, Figure S1: A similarity matrix showing the RMSD generated from the ‘all-against-all’ comparison of the CAT domains of PARP1, PARP2, and PARP3, Figure S2: An alignment of the CAT and WGR domains of PARP1, PARP2, and PARP3.

## Abbreviations

αA-F	α-helices A to F from HD
ADP	ADP-ribose
ADP	Adenosine diphosphate
ADPr	ADP-ribosylation
AFM	Atomic force microscopy
ALC1	Amplified in liver cancer 1
AP site	Apurinic/apyrimidinic site
ARH	ADP-ribose hydrolase
ART	ADP-ribosyl transferase
ATM	Ataxia telangiectasia mutated
BER	Base excision repair
BRCA1/2	Breast cancer
BRCT	BRCA1 C-terminal domain
CAT	Catalytic domain
CHD2	Chromodomain-helicase-DNA-binding protein 2
CHD4	Chromodomain-helicase-DNA-binding protein 4
Cryo-EM	Cryo-electron microscopy
DDB1/2	DNA damage binding proteins 1 and 2
DDR	DNA damage response
D-loop	Donor loop of the active site
DNA	Deoxyribonucleic acid
DNAPKcs	DNA protein kinase catalytic subunit
DSB	Double-strand break
ETD	Electron transfer dissociation
FACT	Facilitate chromatin transcription
gap	Missing nucleotide, form of DNA break
GG-NER	Global genome nucleotide excision repair
H1	Linker histone
H2A, H2B, H3, H4	Core histones
HD	Helical domain
HDX-MS	Hydrogen/deuterium exchange-mass spectrometry
HeLa	Cervical cancer cell line from Henrietta Lacks
HPF1	Histone PARylation factor 1
HR	Homologous recombination
HYE	Conserved histidone, tyrosine, glutamic acid motif
IC_50_	Half maximal inhibitory concentration
K_d_	Dissociation constant
MAR	Mono (ADP-ribose)
MARylation	Mono-ADP-ribosylation
MRE11	Meotic recombination 11
MS	Mass spectrometry
NAD^+^	Nicotinamide adenine dinucleotide
NHEJ	Non-homologous end joining
NMNAT-1	Nicotinamide/nicotinic acid mononucleotide adenylyltransferase 1
NMR	Nuclear magnetic resonance
NOESY	Nuclear overhauser effect spectroscopy
NuRD	Nucleosome remodeling and deacetylase
p53	Tumour suppressor named after its apparent MW on SDS-PAGE gel, 53 kDa
PAR	Poly (ADP-ribose)
PARG	Poly(ADP-ribose) glycohydrolase
PARP1	Poly (ADP-ribose) polymerase 1
PARP2	Poly (ADP-ribose) polymerase 2
PARP3	Poly (ADP-ribose) polymerase 3
PARPi	PARP inhibitor
PARylation	Poly-ADP-ribosylation
PDB	Protein data bank
PTM	Post-translational modification
RMSD	Root mean square deviation
RNA	Ribonucleic acid
SAP	SAF/acinus/PIAS
SNF2	Sucrose non-fermenting protein 2
SSB	Single-strand break
TDP1	Tyrosyl-DNA phosphodiesterase 1
Top1cc	TOP1 cleavage complexes
V(D)J	Variable, diversity and joining
WGR	Conserved tryptophan, glycine, arginine-motif domain
XRCC1	X-ray repair cross complementing group 1
Zn1	Zinc finger 1
Zn2	Zinc finger 2
Zn3	Zinc finger 3
