# Supplementary material for: PARP Power: A Structural Perspective on PARP1, PARP2, and PARP3 in DNA Damage Repair and Nucleosome Remodelling
Source: Int J Mol Sci. 2021 May 12;22(10):5112. doi: 10.3390/ijms22105112 (PMC8150716; doi:10.3390/ijms22105112)
Supplement: Supplementary file 1 [file ijms-22-05112-s001.zip › ijms-1193536-supplementary.pdf]

Supplementary Figures

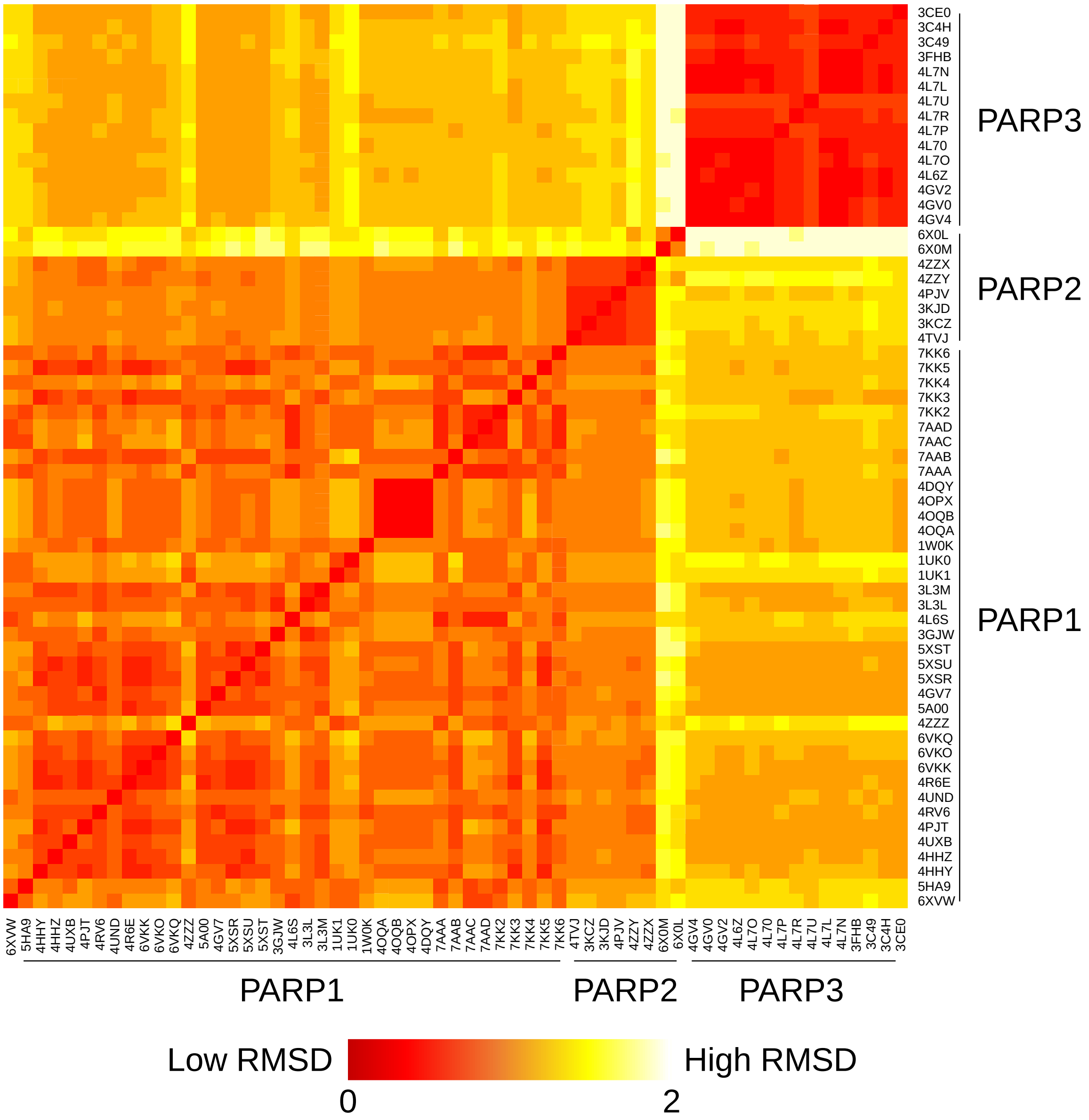

Supplementary Figure 1. A similarity matrix showing the RMSD generated from the 'all-against-all' (Copyright (c) 2004 Robert L. Campbell [1]) comparison of the CAT (ART and HD) domains of PARP1, PARP2, and PARP3 from published sources (PDB). Red represents a low Cα RMSD and yellow a higher RMSD of 2.

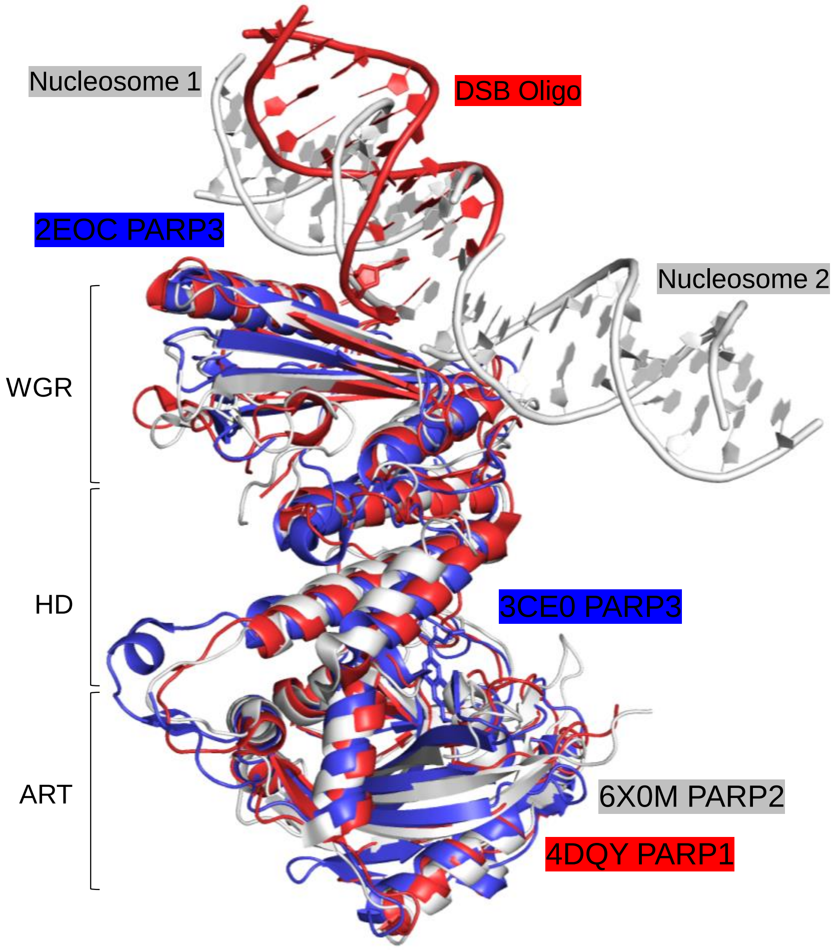

***Supplementary Figure 2*. An alignment of the CAT and WGR Domains of PARP1, PARP2, and PARP3.** Structural superposition and figure were made in PyMOL [2]. PARP1 (red, PDB 4DQY [3]) is taken from a crystal structure of PARP1 bound to one side of a DSB model. PARP2 (white, PDB 6X0M [4]) is taken from a cryo-EM structure of PARP2 bound to HPF1 bridging a DSB between two nucleosomes. The WGR domain of PARP3 is a solution NMR structure (blue, PDB 2EOC) and the CAT domain is a crystal structure (blue, PDB 3CE0 [5]).

References

1. Campbell, R. 2004. Downloaded from http://pldserver1.biochem.queensu.ca/~rlc/work/pymol/.
2. *The PyMOL Molecular Graphics System*; Version: 2.4.2; Schrödinger, LLC: New York, NY, USA, 2021.
3. Langelier, M.-F.; Planck, J.L.; Roy, S.; Pascal, J.M. Structural Basis for DNA Damage-Dependent Poly(ADP-Ribosyl)Ation by Human PARP-1. *Science* **2012**, *336*, 728–732, doi:10.1126/science.1216338.
4. Bilokapic, S.; Suskiewicz, M.J.; Ahel, I.; Halic, M. Bridging of DNA Breaks Activates PARP2–HPF1 to Modify Chromatin. *Nature* **2020**, *585*, 609–613, doi:10.1038/s41586-020-2725-7.
5. Lehtiö, L.; Jemth, A.-S.; Collins, R.; Loseva, O.; Johansson, A.; Markova, N.; Hammarström, M.; Flores, A.; Holmberg-Schiavone, L.; Weigelt, J.; et al. Structural Basis for Inhibitor Specificity in Human Poly(ADP-Ribose) Polymerase-3. *J. Med. Chem.* **2009**, *52*, 3108–3111, doi:10.1021/jm900052j.
